# Editors’ Book Table

**Published:** 1873-10

**Authors:** 


### Editors' Book Table.

[NOTE. — All works reviewed in the columns of the CHICAGO MEDICAL JOURNAL may be found in the extensive stock of W. B. KEEN, COOKE & Co., whose catalogue of Medical Books will be sent to any address upon request.]

BOOKS RECEIVED.

*Report of the Columbia Hospital for Women and Lying-in Asylum, Washington, D. C.* By J. HARRY THOMPSON, A.M., M.D., Surgeon-in-Chief. With an Appendix. Washington: Government Printing Office. 1873.

One of the most valuable contributions to gynæcological medicine extant, comprising the results of the treatment of over eleven thousand cases of various phases of the maladies incident to womanhood, together with copious appendices, consisting of reports from the departments of the Diseases of Children, and of Diseases of the Eye and Ear.

The establishment and maintenance of the Hospital whose "Report" is before us, is a gratifying evidence of the appreciation of our representatives in Congress of their duties in regard to the care of residents of every portion of the country, and indeed of the world, who, having been attracted to Washington by their business relations to the General Government, and being prostrated by disease would have become a tax upon the citizens of the District of Columbia, but for this most humane and enlightened provision for their necessities.

The list of beneficiaries of this national charity comprises residents of twenty-four States and twelve foreign countries, a diversity of origin and constitutional peculiarities complicating morbid conditions, but at the same time adding value to the result. The surgeon-in-chief appears to have been peculiarly successful in his operations, many of which have presented unusual complications and difficulties.

The results of the treatment of uterine cancer have been especially gratifying.

The mode of reporting cases in detail imparts to the work a practical value totally unattainable by the statistical method. The work is copiously illustrated by plates, many of which are from photographs, and its mechanical execution reflects credit upon the government printing office from which it emanates. H.

*The Physiology and Psychology of the Brain.* By HORATIO R. BIGELOW, ESQ., Boston, Mass.

A small pamphlet containing nothing particularly new, being a feeble effort to argue God out of existence. As a specimen of the style of argument designed to accomplish this end, we quote:

"The present tendency is to look upon mind as the force generated by the chemical re-actions in the vesicular neurine of the cortical and hemispherical cells of the brain." * * * * "The will is a product of the mind, and hence, as a dependent force, the paradox of free will becomes apparent." * * * * "And just as the hand by constant use may become palsied, so the mind by the constant inspection of this one idea, breeds unhealthy chemical action in the cells of the brain, and originates," etc., etc. That is to say, the mind is a force generated by chemical action—the mind breeds unhealthy chemical action, *i. e.*, the mind is generated by its results, or the causes of mind result from mind.

The publication of a little more of this sort of logic, will certainly tend to induce the conviction that some minds are generated by chemical action, and that of a very unhealthy sort. H.

*Body and Mind.* An Inquiry into their Connection and Mutual Influence, specially in reference to Mental Disorders: an Enlarged and Revised Edition, to which are added Psycological Essays. By HENRY MAUDSLEY, M.D., Fellow of the Royal College of Physicians; Professor of Medical Jurisprudence in University College, London, etc. London: McMillan & Co. 1873.

So rich a literary and intellectual treat as is supplied by the perusal of this little book, rarely falls to the lot of the medical student, and we shall mark the day devoted to it with a white stone.

The author is a master of physiology, and presents that of the central nervous system in a manner as attractive as instructive. He is certainly entitled to the thanks of all investigators in the higher regions of physiology, for having formulated in language some of the ultimate facts in nervous physiology, which, while recognized as truths by some, are, to the many, vague, indistinct, and altogether "without form and void."

The author pushes his investigations into the nature of mental functions and processes from a physiological stand-point, rejecting altogether metaphysical data as bases for conclusions, and we cannot help feeling that he is a little too exclusive in this rejection of all *a priori* conceptions.

There appears to be no essential difference between the theory of the creation of what is termed mind as an entity, and of a nerve-cell which is claimed as the agent of its production.

Whether the investigator proceed upon the theory of progressive development of organisms, or of specific creation, he must, sooner or later, reach a limit beyond which his inductions will not carry him, here he must stop, even the theory of spontaneous generation will not aid him; "*ex nihilo nihil fit*," in the natural order. While we do not presume to insinuate that so great a mind as that of Maudsley could be tainted with atheism, such is, nevertheless, the necessary result of these development theories, if they are pushed to their logical consequences, of which he very prudently stops short.

The critical essays in the latter part of the book are extremely fascinating. The character of Hamlet is analyzed in a masterly manner, the treatment of the subject proclaiming its author to be as much at home in the realm of belles-lettres, as in those of physiology and psychology. H.

*Skin Diseases, Their Description, Pathology, Diagnosis and Treatment.* By TILBURY FOX, M.D., London, Fellow of the Royal College of Physicians of London; Physician to the Department for Skin Diseases in University College Hospital; Fellow of University College. Second American, from the Third London Edition; Rewritten and Enlarged, with a Cutaneous Pharmacopœia, a Glossarial Index, and Sixty-seven Additional Illustrations. New York: William Wood & Co. 1873.

"To be a successful dermatologist, it is necessary to be a well informed physician," says the author of the above named treatise, and its careful examination will convince the student that it is the work of one who can lay claim to both these distinctions. The earlier chapters upon the method of study, upon the anatomy and anatomical consideration of the skin and its appendages, upon its general pathology, etiology and therapeutics, are especially well systematized and lucid. The chapter on classification presents sound, new and meritorious ideas, and the glossarial index will be found a great convenience to the busy practitioner. The descriptions of the less common forms of disease are numerous and from reliable sources. The work is profusely illustrated with woodcuts—many of which are new. H.

*The Cerebral Convolutions of Man;* represented according to Original Observations, especially upon the Development in the Fœtus. Intended for the use of Physicians. By ALEXANDER ECKER, Professor of Anatomy and Comparative Anatomy, in the University of Freiburg, Baden. Translated by ROBERT T. EDES, M.D. New York: D. Appleton & Co. 1873.

A laborious and faithful analysis of the lobes, convolutions, and fissures of the brain, aptly illustrated with diagrams in outline, which cannot fail to be instructive to the diligent reader. The author has been a faithful student of the literature of his subject, which being for the most part in French and German, is inaccessible to the majority of American readers. The translator has done his work with remarkable fidelity, and has demonstrated his knowledge, not only of German, but, what is still more rare, of accurate English. H.

*Epidemic or Malignant Cholera.* By ALFRED STILLE, M.D., Professor of Theory and Practice of Medicine in the University of Pennsylvania. Reprinted from the Philadelphia Medical Times. Philadelphia: J. B. Lippincott & Co. 1873.

A brief summary of the history, etiology, pathology and treatment of this formidable malady, from the pen of one of the most accomplished members of our profession. The reader will be disposed to find fault with the brevity of the pamphlet, and regret that the doctor has not entered more fully into the details of treatment. It is satisfactory to know that he repudiates the theory of the common origin of cholera, and claims for it a specific origin and an invariable source.

Those who are doubtful upon this head, should dispel their doubts by the careful perusal of this little monograph, in which their time will be by no means wasted. H.

*Mineral Springs of North America*—How to Reach and How to Use Them. By J. J. MOORMAN, M.D., Physician to the White Sulphur Springs; Prof. of Medical Jurisprudence and Hygiene in Washington University, Baltimore, etc., etc. Philadelphia: J. B. Lippincott & Co. 1873.

*The Mineral Springs of the United States and Canada*, with Analyses and Notes on the Prominent Spas of Europe, and a List of Sea-Side Resorts. By GEO. E. WALTON, M.D., Lecturer on Materia Medica in the Miami Medical College, Cincinnati; Committee of the Medical Association of the State of Ohio, on "Therapeutics of Mineral Springs." New York: D. Appleton & Company, 549 & 551 Broadway. 1873.

